# Simulated target search by bats using biomimetic SCAT biosonar model

**DOI:** 10.3389/fncom.2026.1805106

**Published:** 2026-04-08

**Authors:** James A. Simmons, Prithvi Thakur, Ashok Ragavendran, Chen Ming, Andrea Megela Simmons

**Affiliations:** 1 Department of Neuroscience, Brown University, Providence, RI, United States; 2 Carney Institute for Brain Science, Brown University, Providence, RI, United States; 3 Center for Computation and Visualization, Office of Information Technology, Brown University, Providence, RI, United States; 4 Department of Cognitive and Psychological Sciences, Brown University, Providence, RI, United States

**Keywords:** biosonar, echo delay, echo spectrum, echolocating bats, target, target image

## Abstract

Echolocating big brown bats broadcast short, wideband ultrasonic FM pulses for foraging and navigation. These broadcasts contain frequencies from 100 to 20 kHz (wavelengths 0.34–1.7 cm). Bats perceive target distance by measuring the time delay between the outgoing pulse and the returning echo. Acuity of this delay perception depends on the frequency content of echoes and the associated microsecond-level coherence between neural representations of the 1st and 2nd harmonic frequencies. Bats perceive target shape by estimating differences in the delay of mini-echoes from different reflecting points, or glints, within the target. A matched-filter receiver would register glints as prominent peaks in the pulse-echo cross-correlation output, but in bats the overlapping glint reflections mix together to create echo interference patterns that are transposed back into delay estimates. The process is modeled as spectrogram correlation and transformation (SCAT). The first, nearest glint is registered by echo delay itself, but subsequent glints are extracted from the nulls in the interference spectrum. Here, the SCAT receiver was evaluated for its ability to locate targets with a specific glint spacing in the 2D range/cross-range plane while rejecting other targets with larger or smaller spacings.

## 1 Introduction

The study of bat echolocation first focused on determining how flying bats could avoid obstacles in the dark (Griffin, 1958; Grinnell et al., 2015; Yovel, 2025). Further field observations and laboratory experiments established that bats also use echolocation to locate and capture flying insect prey (Griffin, 1953; Griffin et al., 1960). Yet more experiments documented that bats could discriminate among several airborne objects—edible mealworms presented along with inedible disks or spheres of similar size (Griffin et al., 1965). Results of these experiments led to questions about the nature of insect echoes (Griffin, 1967; Simmons and Chen, 1989; Moss and Zagaeski, 1994) and about how the bat’s auditory system might distinguish insects from other objects based on the neural representation of echo information (Simmons et al., 1995). Echoes from insects contain glints or mini-reflections, peaks and nulls in the echo spectrum, from prominent body parts, such as the head, wings, and abdomen (Griffin, 1967; Simmons and Chen, 1989). Glints return individual mini-echoes that merge together at the bat’s ear to form a composite sound with a delay associated with target distance (5.8 ms per meter of target range) and a spectrum determined by the time separations between the glint reflections (Griffin, 1967). Non-prey objects also return echoes that contain several glint-like reflections (Simmons and Chen, 1989). How bats perceive glints and discriminate between edible and non-edible objects has been investigated in big brown bats (*Eptesicus fuscus*), an insectivorous species that broadcasts short, downward-sweeping frequency-modulated (FM) pulses spanning frequencies from 100 to 20 kHz (wavelengths of 1.7–0.34 cm). The critical feature for auditory representation of glints is the integration time for echo reception in the bat’s inner ear, which is about 350 μs (Au and Simmons, 2007). The first mini-reflection evokes neural responses that register its time-of-occurrence relative to the responses evoked by the broadcast; this time difference marks echo delay and thus target range (Sanderson and Simmons, 2000; Sanderson and Simmons, 2003; Sanderson and Simmons, 2005). If other mini-reflections arrive within the integration time window, they interfere with each other to form patterns of spectral nulls separated in frequency by the inverse of their time separation (Simmons et al., 1995).

The airborne targets used in early behavioral experiments (mealworms, disks, spheres) (Au and Simmons, 2007) had linear dimensions up to ∼2 cm, creating delay separations up to about 100 μs (Simmons and Chen, 1989). Consequently, the glint reflections all arrive within the inner ear’s integration time and produce interference spectra that are encoded as amplitude levels across auditory neurons tuned to ultrasonic frequencies in the broadcasts (Simmons et al., 1996). These spectra represent the arrangement of glints in the target, and thus target shape. The surprising result from psychophysical experiments on echo-delay discrimination by big brown bats is that both the overall echo delay (for target range) and the delay spacing of glint reflections (for target shape) are perceived as *delays*, even though only the first glint is registered as delay itself (Simmons et al., 1995). The additional glints are registered by interference nulls in the spectrum, and not directly by their individually delayed reflections. The bat’s auditory system transposes the frequencies of nulls in the interference spectrum back into the time intervals between glint reflections (Sanderson and Simmons, 2003; Simmons et al., 1996). That is, both target range and target shape are perceived spatially in terms of distance from the bat (Simmons et al., 1995; DeLong et al., 2008).

**FIGURE 1:**
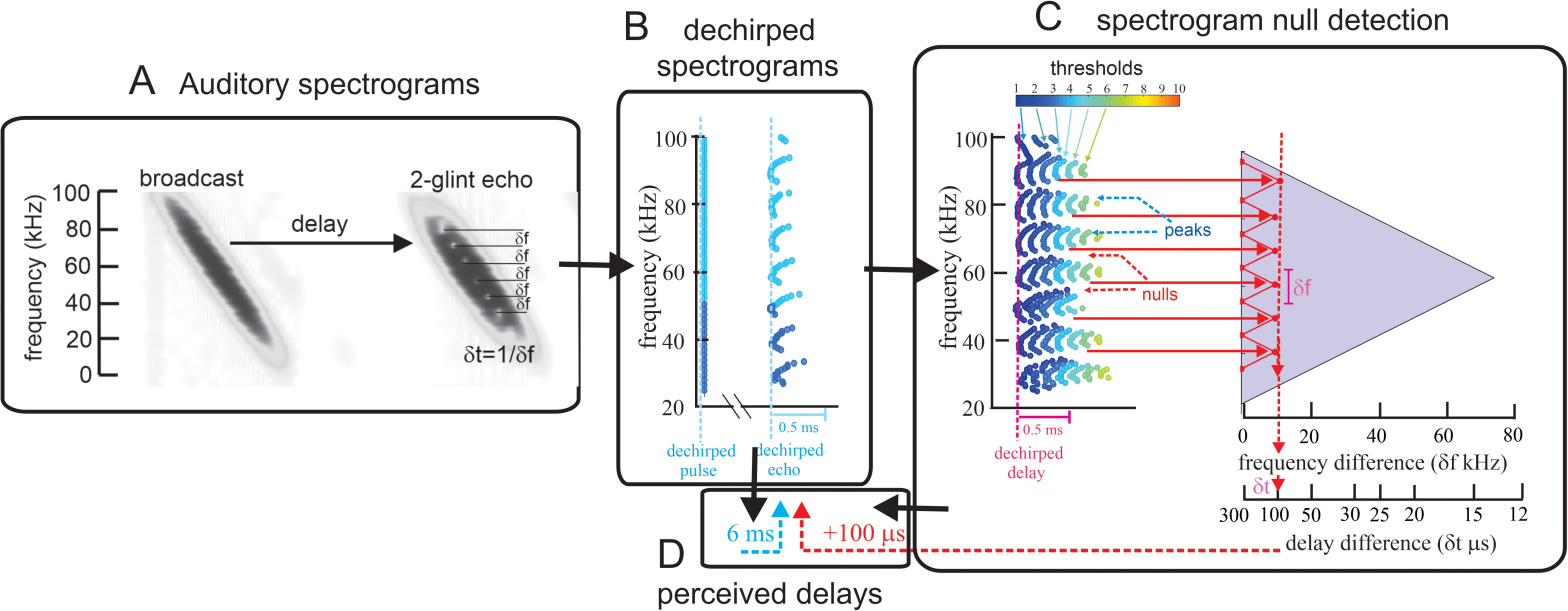
Explanation of SCAT model (Ming et al., 2021). **(A)** Spectrogram (left) of model FM biosonar broadcast sweeping down from 100 to 20 kHz, followed by 2-glint echo (right) at a delay of 6 ms (1 m target range). Glints are 100 μs apart, equivalent to 17 mm separation in the target. The integration time of spectrograms (width of dark ridges) is about 350 μs, so the glint reflections mix together to create spectral interference nulls 10 kHz apart (δf = 1/δt). **(B)** Initial stage of processing uses bandpass filters to segment FM sweeps into parallel, overlapping bands. Traces made of blue dots representing one threshold show the time-of-occurrence of detection events across frequencies. The FM sweeps are dechirped to bring different frequencies and threshold events for the broadcast to the same time-of-occurrence. In contrast, thresholds for echoes are spread out due to amplitude-latency trading, which retards events when amplitude decreases. The threshold events form a scalloped pattern with the center frequencies of the nulls registered at the right-side peaks (i.e., down-time) of the wavy pattern. The overall 6 ms delay of the echo is represented by the displacement of the echo thresholds to a later time than the broadcast thresholds. **(C)** The network of delay registrations for different threshold levels shows the full scalloped pattern from B, but with all 10 thresholds included. The use of single time events to mark each frequency and threshold level reduces the computations to large numbers of pulse-to-echo time-intervals that progress to slightly longer delays as the threshold increases. The gray triangle contains networks of connections across all frequencies at different fixed frequency separations. For any given frequency spacing (e.g., red zig-zag line), the locations of the right-hand tips of the scalloped pattern marks the vertical frequency spacing of the nulls along the horizontal axis of the triangle. Larger null-frequency spacings from shorter glint delay spacings project the tips of the zig-zag farther to the right, which marks the glint spacing on the two horizontal axes (δf = 1/δt). **(D)** The completed SCAT image combines the overall echo delay derived from the dechirped spectrograms in B with the vertical alignment of the zig-zag tips indicated the delay spacing of the glint reflections.

The dual process of representing target range from echo delay and target shape from the transposed echo spectrum is modeled as Spectrogram Correlation and Transformation (SCAT) (Saillant et al., 1993; Ming et al., 2021). Figure 1 is a graphic explanation of the SCAT process. A conventional sonar receiver uses pulse-to-echo cross-correlation to represent targets (Skolnik, 1980; Denny, 2007), which results in accurate display of the target’s range and shape. Each mini-reflection, from the first glint followed by any other mini-reflections, is displayed by its own peak in the function. All of the glint delays are estimated by a single signal processing algorithm, so they are not compartmentalized as range or shape, just as delays. The SCAT receiver yields a similar display after the echo spectrum is transposed back into the delay separations of the glint mini-reflections. First, overall echo delay is estimated by spectrogram correlation, which is a frequency-by-frequency estimate of delay pooled across all frequencies (Figures 1A,B; Suga et al., 1990). Then, the locations of spectral nulls distributed across the spectrum are extracted and displayed to invert the frequency spacings of the nulls into estimated glint delay spacings (Figures 1B,C). The resulting compound SCAT equivalent to cross-correlation is portrayed pictorially in this output. We emphasize that this image is our vision-oriented display; the auditory process internal to the bat unfolds in the time domain across numerous frequency channels to yield the bat’s percept.

There are certain computational advantages that accrue to SCAT reception. For example, target range, which is expressed in meters, comes from spectrogram correlation, whereas target shape comes from spectrogram transformation expressed in millimeters. The numerical values for glint delay are expressed relative to the arrival time of the first (nearest) glint reflection. Subsequent glint delays are perceived in relation to this value, making target shape a separate computational compartment from target range. Another advantage derives from the distribution for different degrees of sharpness for frequency tuning to each frequency in the FM sweep of an echo. The range of tuning widths spans from about 15 to 0.5 kHz, with tuned frequencies from 10 to 90 kHz (Sanderson and Simmons, 2000; Ferragamo et al., 1998). This allows detecting individual spectral nulls caused by the delay separation of glint reflections around 100 μs, but the absence of wider tuning widths means that attenuation over a broad frequency band, particularly from lowpass filtering of echoes, is not registered as a single null. Instead, it has to be registered by all of the potential nulls that fit into the wider spectral region of attenuation. The proliferation of detected nulls leads to corresponding perception of the proliferation of the glint delays that these nulls represent, which results in improved suppression of off-axis clutter (Bates et al., 2011; Simmons, 2014; Warnecke and Simmons, 2016; Shriram and Simmons, 2019). We undertook the simulations described here to establish methods for evaluating these ancillary advantages to SCAT in practical situations.

## 2 Materials and methods

**FIGURE 2:**
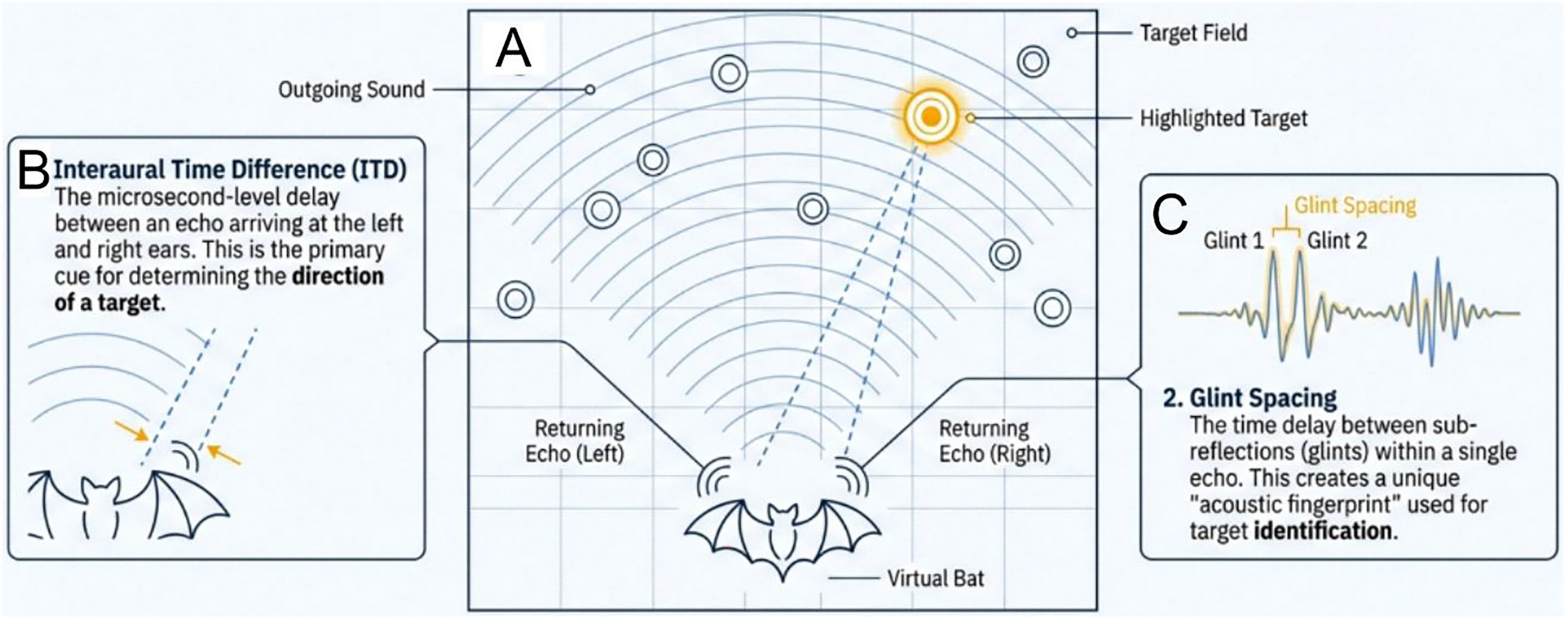
Diagrams showing the SCAT receiver used to find and classify simulated 2-glint sonar targets distributed on the range-crossrange plane (Batnav routines). **(A)** Model “bat” emits FM biosonar signal (Figure 1A) that spreads over plane to impinge on objects located at different target ranges and in different horizontal directions (azimuths). Targets contain two reflecting glints; the desired target has 100-μs glint delay spacing (orange) while other targets have shorter or longer spacings. Receiver determines distance from echo delay (Figure 1B), initially orienting to and approaching the nearest target using sonar beam aim to focus on target while turning in “flight” to approach nearer. Azimuth is determined from echo arrival-time differences at the left and right “ears.” **(B)** Binaural echo delay differences drive both beam aim and flight direction toward the selected (nearest) target. **(C)** Target returns two closely-spaced reflections from target’s glints, which mix together within the receiver’s integration-time to create interference nulls distributed along spectrogram’s frequency axis (Figure 1A). The SCAT transformation displays the delay spacing of the glint reflections on a schematic representation of both target range and echo glint separation (Figure 1C). The model bat continues its approach until it had determined whether the glint spacing is 100 μs, which results in capture, or is not 100 μs, which results in target rejection and a new approach to the next nearest target.

**FIGURE 3:**
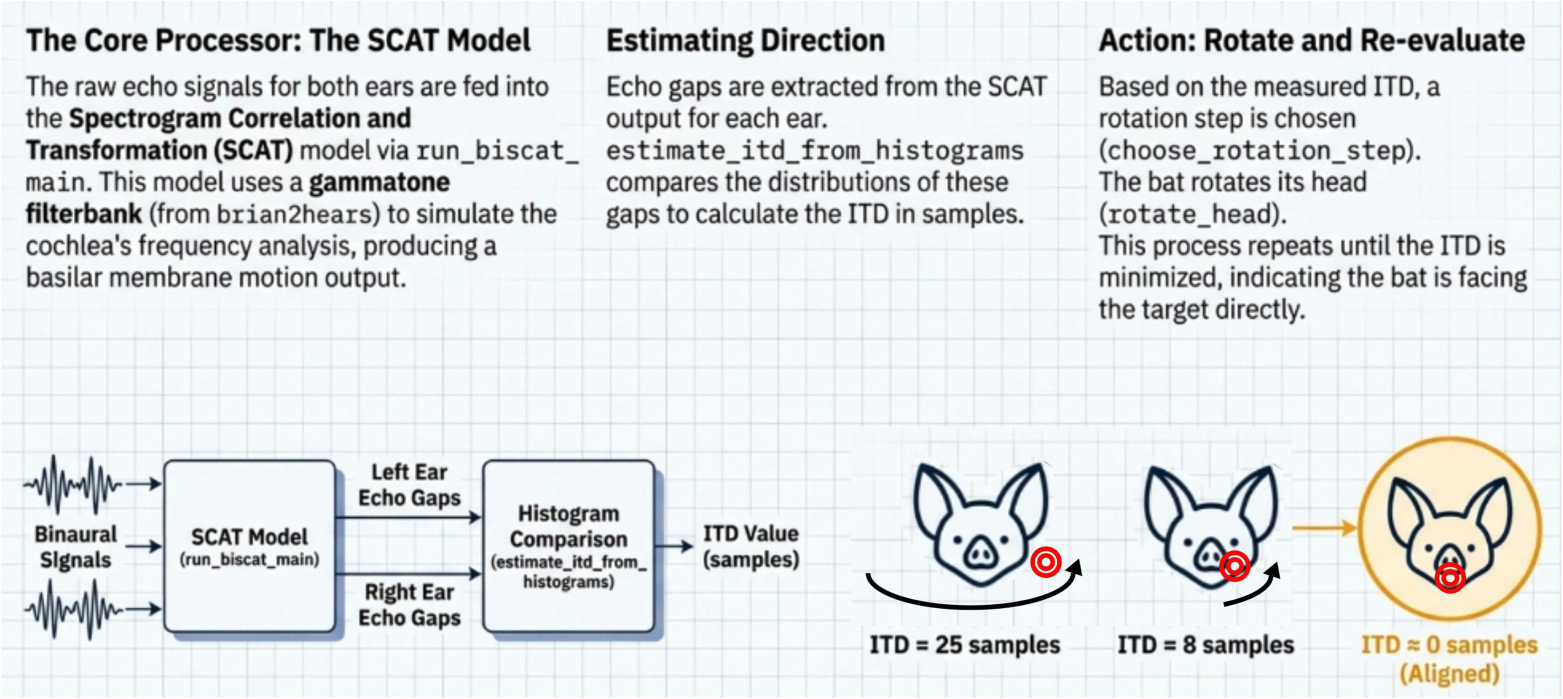
The Batnav simulation runs in successive time epochs marked by the emission of each FM broadcast. The two-glint echo from the nearest target is received at the left and right ears and the target’s azimuth is determined from the binaural delay difference. Both beam-aim and flight direction are changed to approach the target. Across successive echoes, the bat’s head rotates to aim at the target, thus reducing and eventually eliminating the target’s offset from straight ahead (placement of succession of red targets at the right part of the diagram). The 2D delay separation of the two glint reflections (from the spacing of the frequency nulls in left and right binaural spectrograms) yields a binaural spectral disparity that signifies the aspect-angle view of the targets two glints. As the target’s azimuth offset relative to the head decreases, this binaural disparity ceases to register both azimuth and target aspect-angle to become only aspect-angle, thus yielding a better approximation for interglint spacing.

The SCAT model of wideband echolocation (Saillant et al., 1993) in its present form (Ming et al., 2021) emulates the formation of biosonar perception using spectrograms of broadcasts and echoes (Figure 1). Here, we employ the SCAT model as a sonar receiver in the task of finding simulated insect-sized 2-glint targets whose locations are distributed on the range-crossrange plane (i.e., distance and azimuth). The simulation procedure, named *Batnav* (Figure 2), mimics the transmission of bat-like FM biosonar pulses to guide the movement of a SCAT receiver toward a succession of model targets in search of a specific target shape, represented as two glints spaced 1.7 cm apart (100 μs when viewed from end-on aspect-angle). The occurrence of each broadcast pulse defines a time-step in the simulation—an epoch in time when the emitted pulses spread out on the plane, impinge on the targets, and return to the bat as “echoes” from different distances and different directions. Each emitted pulse sweeps linearly downward from 100 to 20 kHz (Figure 1A). The “echo” from each target contains two mini-reflections that arrive at the model bat’s ears with a time separation defined by the glint spacing itself and the aspect view of the target. The “bat” begins to aim its sonar in the direction of the nearest target (shortest echo delay), moves forward toward this target while turning, and then emits the next broadcast. The approach process consists of a succession of broadcast epochs with their associated progressive approximations of the target’s direction with both flight path and sonar beam aim (Figure 2). The method, summarized in Figure 3, creates a 2D range-crossrange (i.e., horizontal) plane, enters a simulated “bat” onto the plane, which then emits broadcasts designed to mimic biosonar pulses to guide movement on the plane in response to various simulated targets. All the targets consist of two glints, but only the desired target has 1.7 cm 2-glint spacing, approximately in the middle of typical glint spacings for insects hunted by big brown bats (Shriram and Simmons, 2019; Clare et al., 2014). The other targets contain two glints separated by 0.51–17 cm (30–1,000 μs). (The SCAT process treats glint-reflection spacings of less than 350 μs as represented by nulls in the spectrogram, while larger spacings are represented as separate echoes, one for each glint.)

**FIGURE 4:**
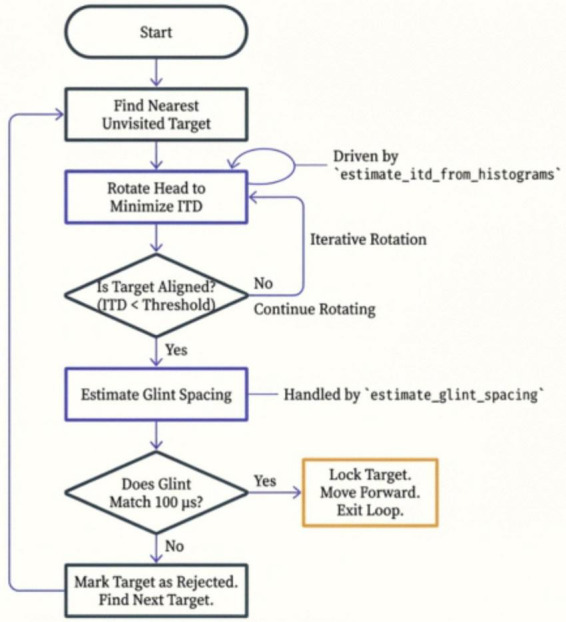
Flow chart for Batnav algorithms. Search process begins with emission of first FM broadcast which leads to detection of the nearest target from the shortest delay of its echo among other echoes acquired in the first broadcast epoch. The flight path and beam aim are adjusted toward this target based on the echo’s binaural echo arrival-time difference. On successive broadcast epochs, the bat approaches nearer and continues to improve its direction of flight and beam aim toward the target. When the binaural echo time difference approaches zero after successive epochs, the glint spacing is estimated with minimal distortion due to misalignment of the beam. The target is either accepted as having a 1.7 cm glint spacing (100 μs reflection spacing) and “captured,” or rejected and the search begins for the next, nearest target.

The Batnav algorithms are implemented in Python and run on the OSCAR supercomputer at the Brown University Computational Biology Core. To accomplish the tasks in Figure 3, these algorithms make a series of detections, actions, and decisions to generate each successive representation of the target’s range, direction, and glint structure for each broadcast epoch (Figure 4). The first broadcast epoch starts the simulation, which finds the nearest target from the shortest delay of its echo. The binaural echo delay difference is used to determine the target’s direction on the 2D plane, which alters flight path by turning toward the target and shifts the sonar beam aim (bat’s head aim) to point more closely at the target. This reduces the binaural delay difference for the next broadcast epoch. Successive broadcast epochs bring the bat nearer to the target and refine the accuracy of beam aim on the 3D plane over successive epochs to “home in” on the target. When aim is adjusted to reduce the binaural delay difference to near zero, the condition is set to provide an estimate of the target’s glint separation. Then, the target is either approached as the correct shape (1.7 cm 2-glint spacing), ending that target’s program loop, or is rejected according to its smaller or larger 2-glint spacing. If not correct, the search resumes to find the next nearest target.

## 3 Results

**FIGURE 5:**
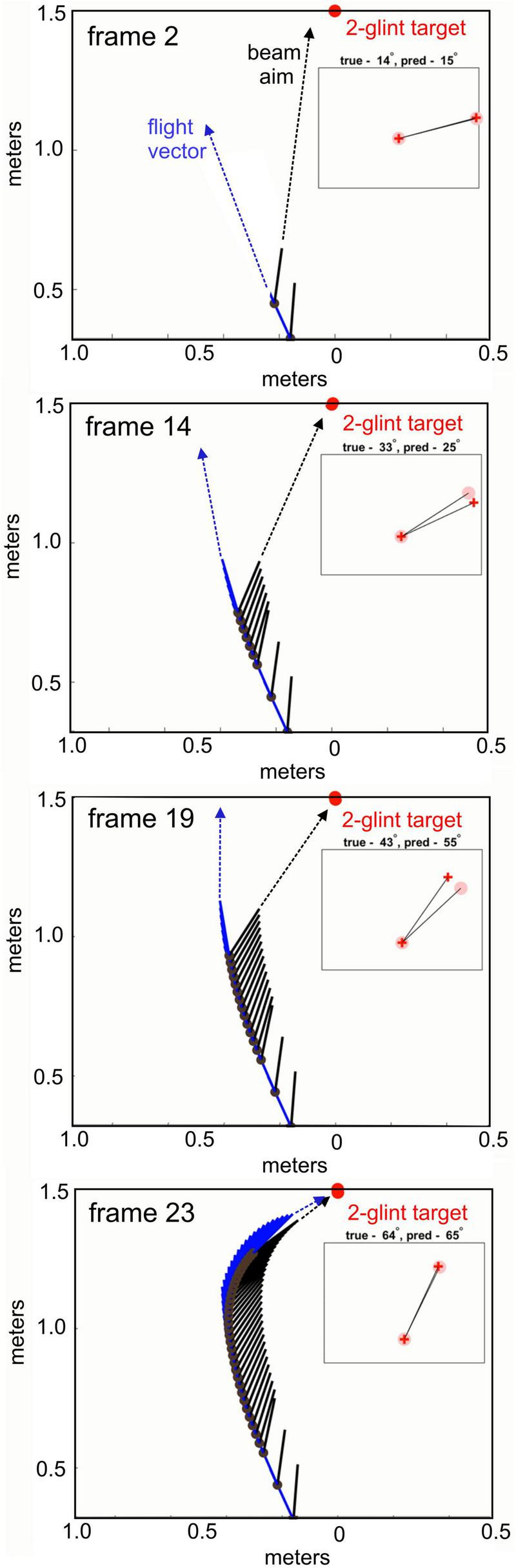
Four frames (top to bottom) from a simulated approach to a single two-glint target (red dot at top) (see Supplementary Video 1). Each frame stands for a broadcast epoch that displays the flight path (blue) and beam aim (black) being updated on reception of the particular 2-glint echo from that broadcast. While beam aim shifts rapidly to fixate on the target’s azimuth, the flight vector moves more slowly to bring the bat onto the target. The inset in each panel shows an approximate orientation of the target’s two glints to be read relative to the aim of the bat’s sonar beam (black arrow). Oriented to the sonar aim vector, it would be a heads-up image in the display. Compartmentalizing the SCAT process separates the image of the two glints for target shape from overall target range.

To illustrate how the simulation works, Figure 5 shows four separated frame images from a run with a single 2-glint target having the desired 1.7 cm glint spacing (animated Supplementary Video 1). In each frame, the target is fixed at the top of the image (red dot) located at zero meters horizontally (crossrange) and 1.5 m vertically (range). Frame 2 (top panel) shows the position of the bat after it enters the scene and has emitted two broadcasts. The flight path and flight direction vector (dark blue arrow and line) traces the bat’s location on the plane and the direction it is heading at this point. The aim of the sonar beam is indicated by the black arrow. The rectangular insert in the frame image shows the display to be read from the bat’s view as the angular orientation of the target (angle values relative to horizontal axis of plane in frame, compare orientation to bat sonar vector). The epoch-by-epoch history of the flight vector and head aim are displayed by the short solid lines that begin at each of the bat’s momentary locations (black dots). Throughout the simulation run, the flight vector lags behind the sonar beam, possibly an effect of inertia, as has been observed in aerial interceptions (Ghose and Moss, 2003; Ghose and Moss, 2006; Ghose et al., 2006). In Frame 14, the bat has progressed through seven more epochs. Its flight continues to track off the target’s direction while keeping the sonar beam locked closely on the target. In Frame 19, after five more epochs, the strategy is clearer, of keeping the beam directly on the target (Masters et al., 1985) while flight curves around to obtain different “looks” from different directions. This gives the model bat a well-defined succession of images of the target as its flight approaches on a different, more circuitous path, so the target appears to rotate in the bat’s view. The effect is to fly part-way around the target to generate a synthetic-aperture reconstruction of its shape from the resulting series of images. Frame 23 shows the bat very close to the target, and the flight path has converged onto the target so both flight and beam aim are similar.

**FIGURE 6:**
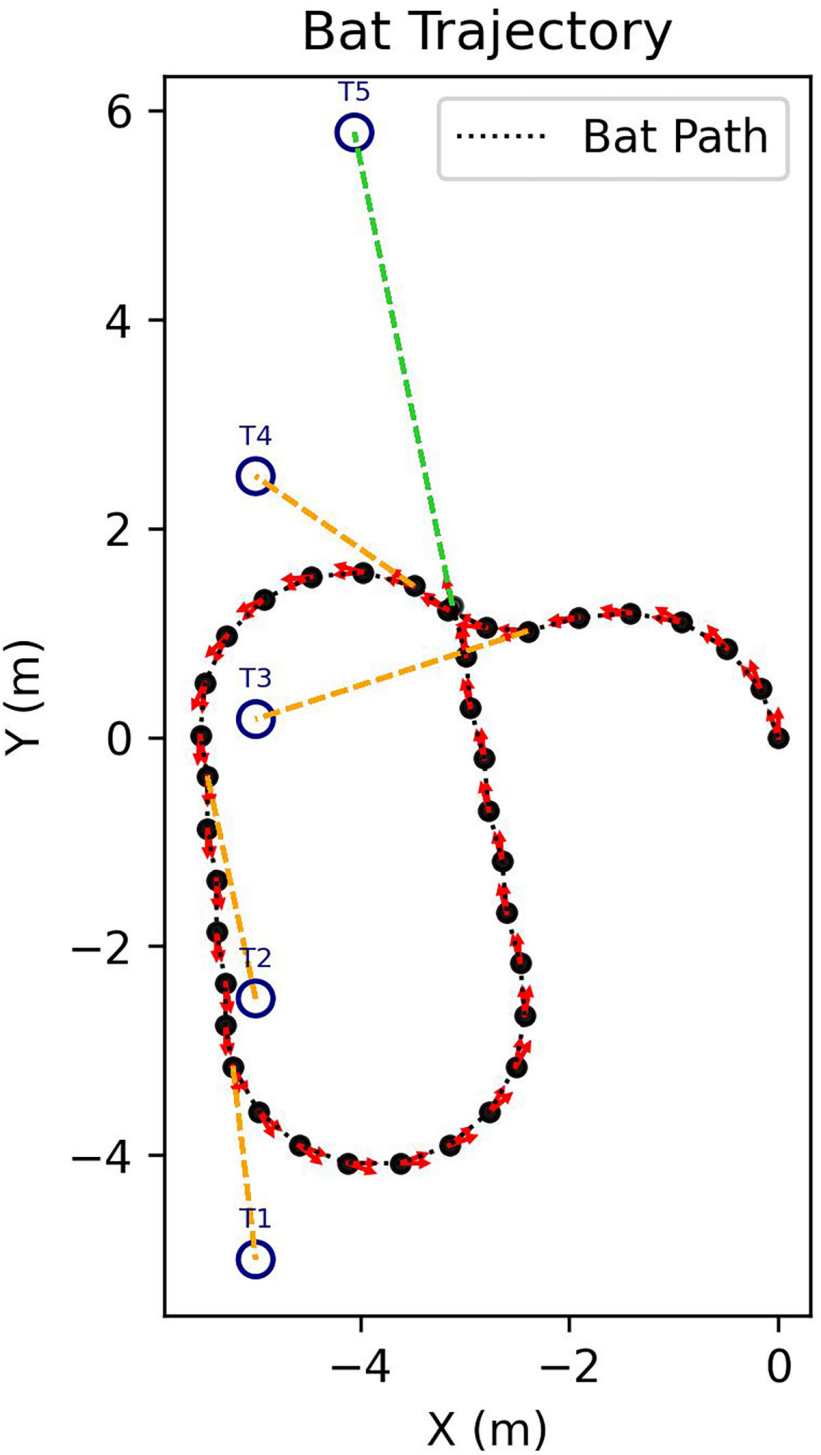
Flight-track history of a simulated search for the 100 μs desired 2-glint target (marked T5) in the presence of four other targets with 50–1,000 μs glint spacings (search starts at XY origin; animation of search in Supplementary Video 2).

**FIGURE 7:**
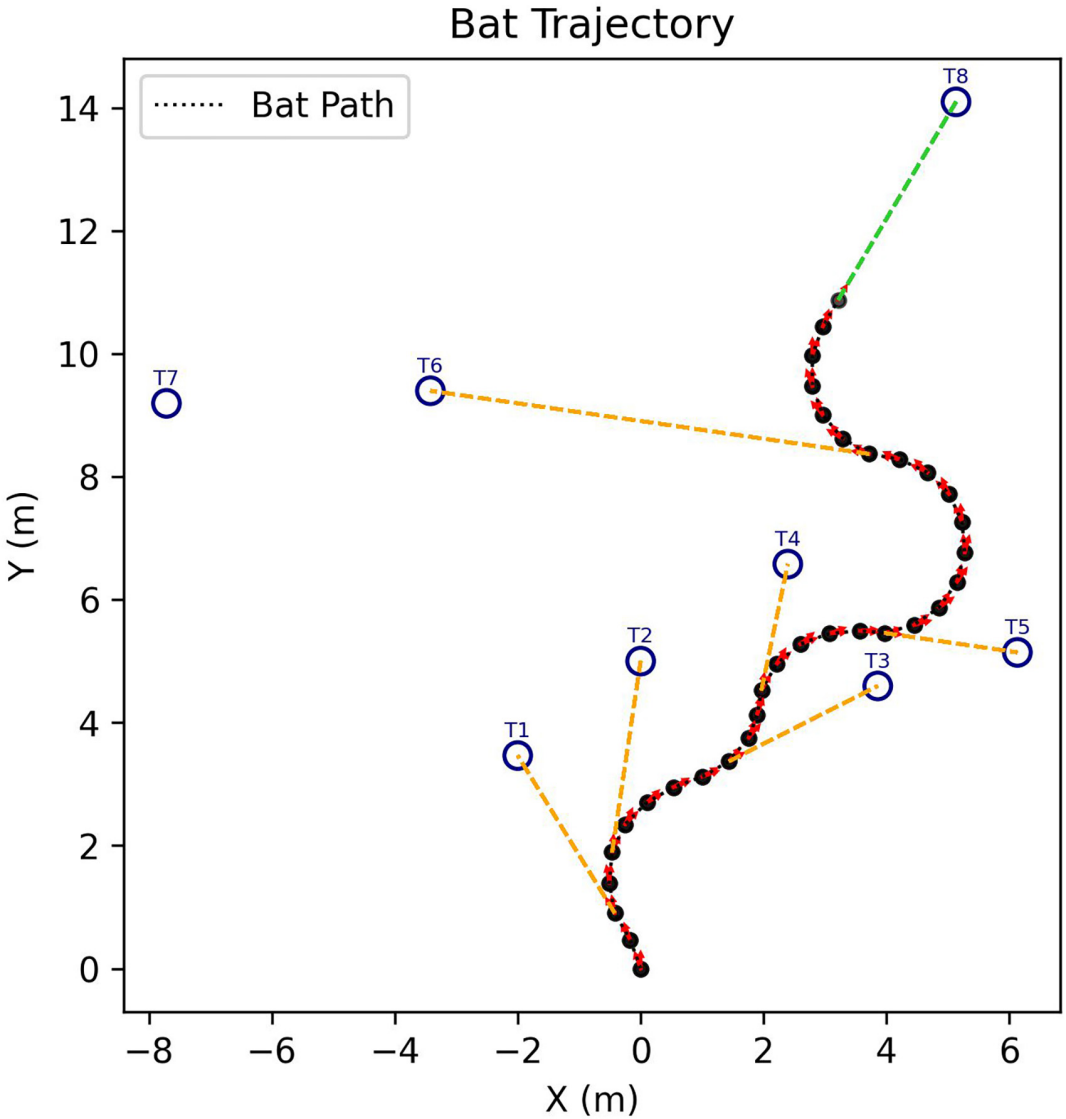
Flight-track history of a simulated search for the 100 μs desired 2-glint target (marked T8) in the presence of seven other targets, one with 300 μs glint spacing and the others with 1,000 μs glint spacings (search starts at XY origin; animation of search in Supplementary Video 3).

**FIGURE 8:**
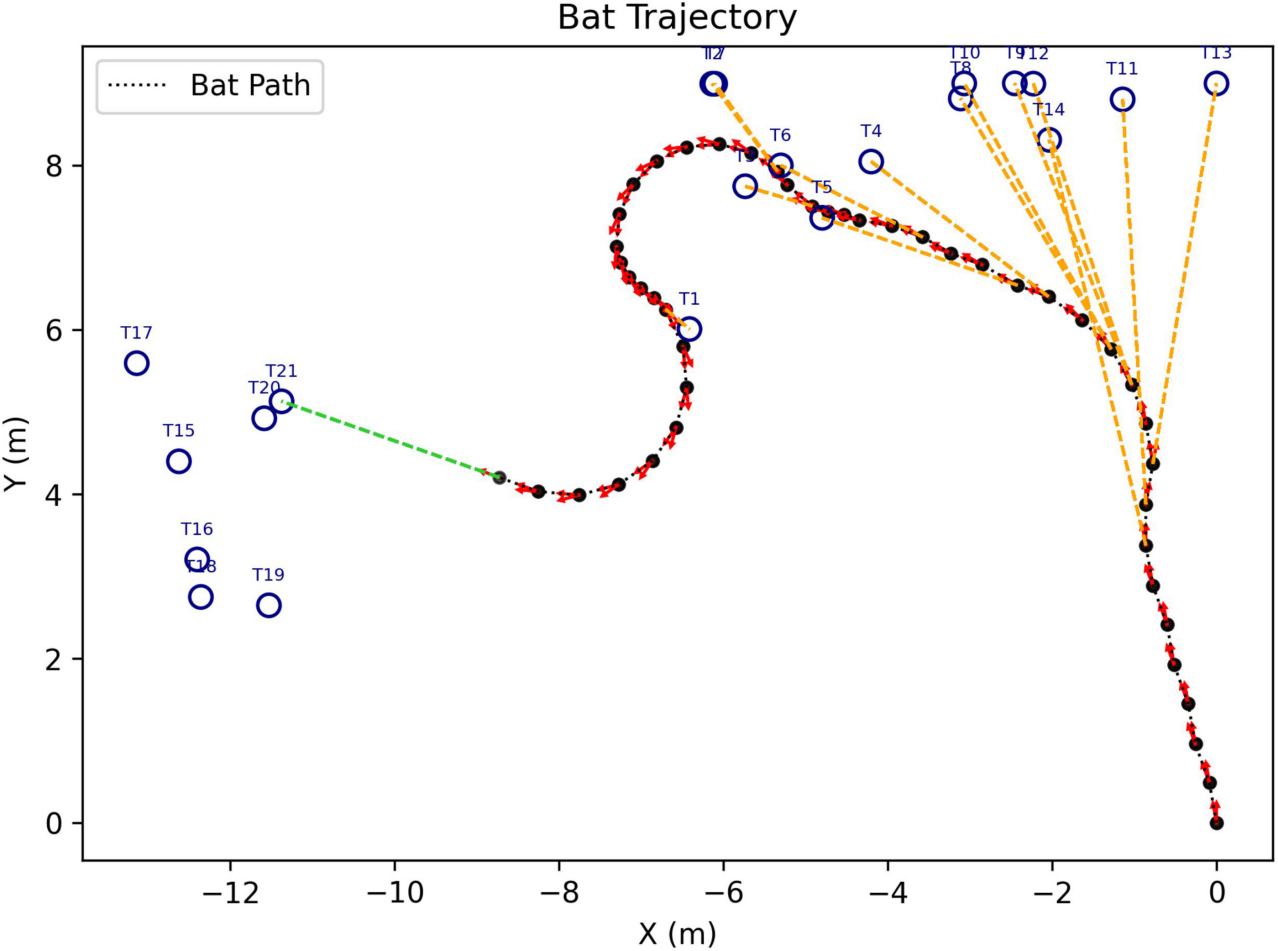
Flight-track history of a simulated search for the 100 μs desired 2-glint target (marked T21) in the presence of 19 other targets with 200–2000 μs glint spacings (search starts at XY origin; animation of search in Supplementary Video 4).

**FIGURE 9:**
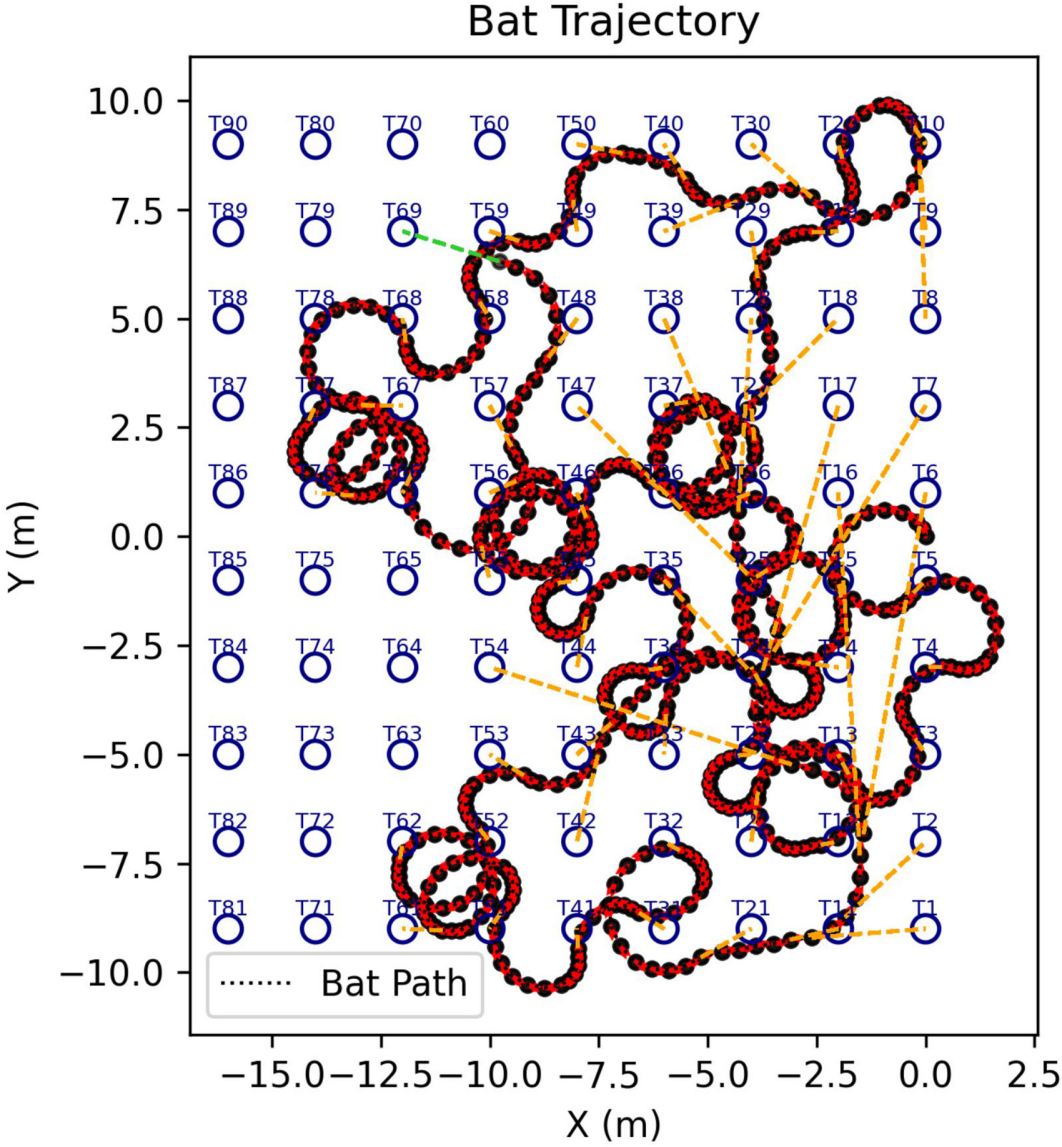
Flight-track history of a simulated search for the 100 μs desired 2-glint target (marked T69) in a grid of 90 targets having multiple glint spacings from 50 to 2000 μs (search starts at XY origin; animation of search in Supplementary Video 5).

The goal is to assess not just how the SCAT receiver might react to an individual target but also how it can search for a specific target (100 μs glint separation) in the presence of multiple targets with different glint separations. In each scene, targets are distributed around the rectangular range (Y) and crossrange (X) plane, and the simulated bat enters at the XY zero origins. Figure 6 shows the model searching a scene containing 5 targets, approaching 4 of them before finding the correct target (summary of tracks in Figure 6; animation in Supplementary Video 2). Note how the flight path is relatively smooth due to a limitation on the rate of turn in flight (see Figure 5). Figure 7 shows a search with eight targets presented; the model searches the scene until it finds and approaches the correct one (animation in Supplementary Video 3). A larger number of targets is illustrated in Figure 8. Twenty targets are distributed along the right and upper sides of the plane, and the simulated bat searches until it locates the 100 μs target (Supplementary Video 4). Finally, in a scene containing 90 targets in a rectangular grid pattern distributed across the range-crossrange plane (Figure 9), the model bat searches with a path that follows numerous circular loops until it locates the correct target (Supplementary Video 5).

**FIGURE 10:**
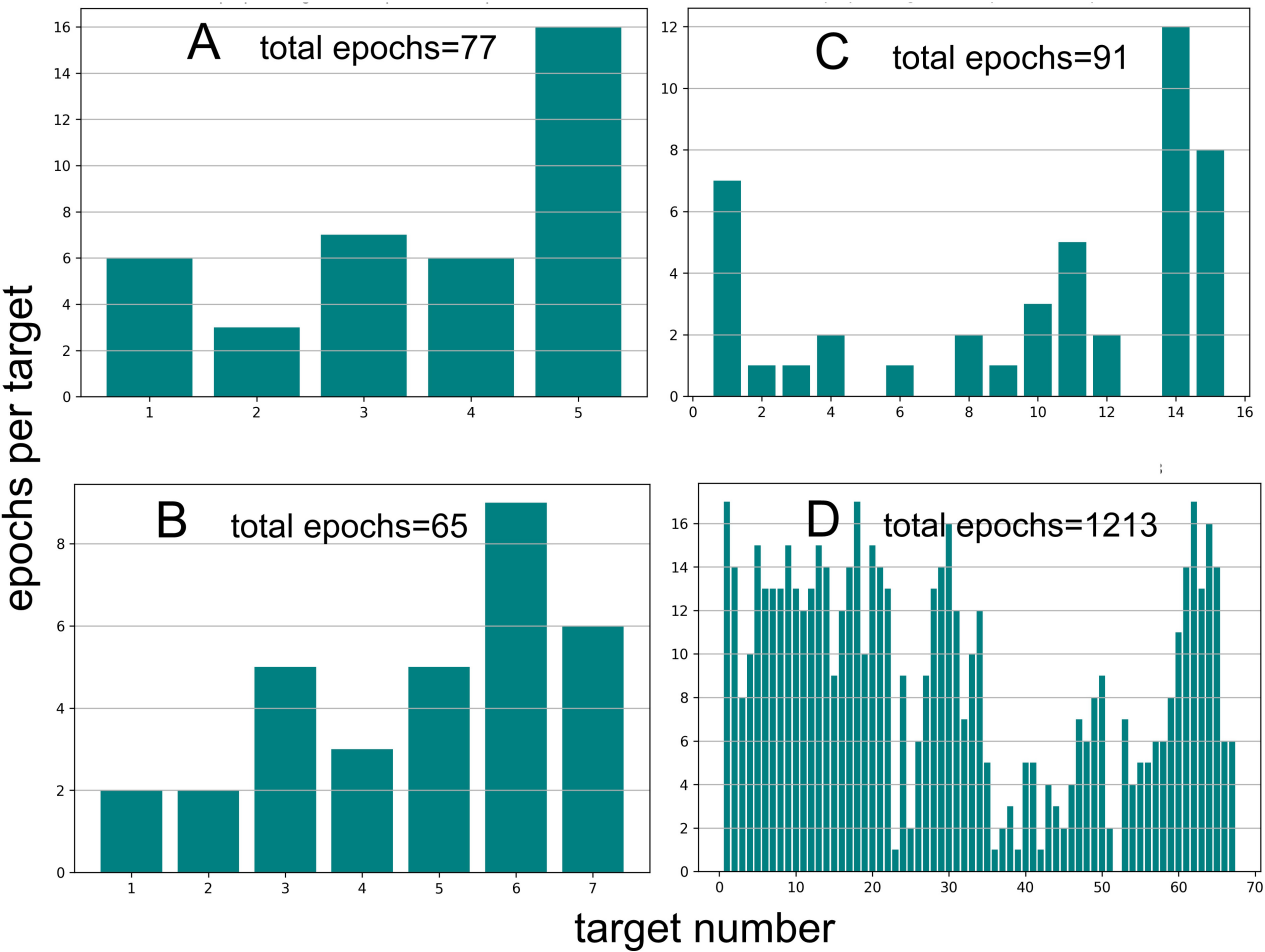
The number of broadcast epochs used to approach successive targets and eventually find the desired 100 μs glint spacing in four scene simulations. Performance measured in epoch numbers is opportunistic, with the placement of individual targets near the starting point (CY origin) being the primary determinant, followed by how subsequent target are located when the first search-and-approach process is completed (**A**, Figure 6; **B**, Figure 7; **C**, Figure 8; **D**, Figure 9).

Figure 10 shows our effort to develop a metric for evaluating the process of finding the correct 2-glint target in different scenes (Figures 6–9). We chose the number of broadcast epochs required to approach and classify each target in succession until the 100-μs glint spacing was found (see sequences in Supplementary Videos 2–5). The total number of broadcast epochs depends most obviously on the size of the target set, which determines the number of targets encountered including the last, correct target. In the four scenes described above, the total number of epochs was 77 (5 targets in Figure 6), 65 (7 targets in Figure 7), 91 (15 targets in Figure 8), and 1,213 (67 targets in Figure 9). The number of epochs for the first target depends on the location of the nearest target to the XY origin, where the model bat begins the simulation. Both the target’s distance and the direction affect performance because the limitation on the rate-of-turn in flight can extend the number of epochs required to approach and aim the sonar beam onto the target to obtain the best estimate of glint separation. The number of epochs required to classify each subsequent target depends on the same factors—distance and direction when the most recent approach has ended with the decision that a target is not the correct one. The simulation then proceeds with variable numbers of epochs per target according to these factors.

## 4 Discussion

In the sonar-scene simulations described here, the SCAT receiver successfully guided searching among different targets to locate a desired 2-glint target with a glint-reflection spacing of 100 μs while rejecting targets with shorter or longer 2-glint separations. The time required to complete the search of each scene was opportunistic; it depended on the number of targets included in the scene and their arrangement relative to the point in the simulation where the bat determined that a given target was not the correct target. When the correct target was found, the simulation ended for that scene. Both distance and direction to the next target affected performance (measured by the number of broadcast epochs required to move to the next target) because the model bat’s rate-of-turn in flight was slower than the speed with which the sonar beam could be turned to fixate the target for the next-most broadcast epoch.

The simulations were as simple as possible: The model only incorporated the bat and the targets in an unbounded rectangular arena as the scene. Critical features of sound propagation in the atmospheric medium, including atmospheric absorption, broadcast beam-shape and receiving directionality, or target reflectivity beyond glint spacing, all of which are frequency-dependent, were not implemented in the simulations. As broadcasts propagate outward from the bat, their amplitude decreases by 6 dB for every doubling of distance. After impinging on targets, each glint reflection similarly decreases on the return back to the bat. Combining outgoing and return travel, echo sound pressure thus decreases by 12 dB per doubling of distance. Additionally, atmospheric absorption is frequency-dependent, increasing from 0.5 dB/m at 20 kHz to over 3 dB/m at 100 kHz. Echoes thus undergo greater attenuation at high frequencies, so they become progressively more lowpass filtered as target range gets longer. The bat’s broadcast and receiving beams sharply focus the full bandwidth of reception only directly to the front, on the axis of the sonar. Beam width decreases at higher frequencies, which excludes more high-frequency content from echoes returned by targets off to the sides, adding yet more lowpass filtering in the echoes. The bat’s SCAT receiver reacts to lowpass echoes by disproportionately inserting spurious glint reflections into perception of echo delay for off-axis targets to reduce the strength of perceived echo delay at the delay of a target located on the sonar beam’s axis (Ming et al., 2021; Warnecke and Simmons, 2016). Real sonar scenes encountered by bats have independently-moving targets with flapping wings, which add time-varying qualities to all of the simulated targets. Insects do not just fly straight, because they take different countermeasures to attacking bats, and these have to be included in more realistic simulations. Often, echoes from vegetation or the ground add clutter to the scene. The directionality of broadcasts and auditory reception play large roles in suppressing interference from clutter, and the distance to different parts of the scene, both the simulated insects and the clutter, affect the spectrum of echoes. Finally, bats frequently hunt in proximity to each other, so individual bats must operate in the presence of sonar sounds emitted by other bats, of both the same and different species (Fenton et al., 2015).

These simulations were undertaken to increase our understanding of how biosonar operates, which necessarily includes understanding what echolocating bats perceive using their active acoustic mode of sensing the environment. Perhaps the greatest long-term challenge in understanding biosonar is that the events depicted in Figure 1 unfold in time-frequency space, not as pictures on the diagrams. The very term “space” indicates that our understanding is too often translated into visual images, which do not constitute our comprehension. The bat’s auditory system represents FM broadcasts and echoes in terms of the times of occurrence of different frequencies and seems unlikely to convert biosonar information into visual percepts. Instead, the percepts are very likely to be retained in an auditory perceptual form.

## Data Availability

The datasets presented in this study can be found in online repositories. The names of the repository/repositories and accession number(s) can be found at: brown-ccv github repo.
